# Seasonal Variation in the Diet Composition of Wild Boars (*Sus scrofa*) Based on Fecal DNA Metabarcoding in Bukhansan National Park, Republic of Korea

**DOI:** 10.3390/ani15243598

**Published:** 2025-12-15

**Authors:** Sanggon Lee, Manh Ha Nguyen, Wonjong Han, Misong Kim, Jiyoung Kim, Euikyeong Kim, Keumchul Shin

**Affiliations:** 1 Department of Forest Environmental Resources, College of Agriculture and Life Sciences, Gyeongsang National University, Jinju 52828, Republic of Korea; tkdrhs170@naver.com (S.L.); gksdnjsb@naver.com (W.H.); misoong427@naver.com (M.K.); 2 Institute of Agriculture & Life Science, Gyeongsang National University, Jinju 52828, Republic of Korea; manhhafsiv@gmail.com; 3 Forest Protection Research Center, Vietnamese Academy of Forest Sciences, Hanoi 11910, Vietnam; 4 Ecological Research Division, Korea National Park Research Institute, Wonju 26441, Republic of Korea; kjy2050@knps.or.kr

**Keywords:** dietary analysis, fecal DNA, macrofungi, metabarcoding, national park, wildlife

## Abstract

The diet of wild boars (*Sus scrofa*) is highly diverse, consisting of both plant and animal materials, and their foraging behavior varies seasonally. However, limited research has investigated the composition of macrofungi in the diet of these omnivorous mammals, and relevant information remains scarce. This study aimed to assess the diversity of plant, animal, and macrofungal components in the diet of wild boars and to evaluate seasonal variations in Bukhansan National Park, Republic of Korea. Fecal samples were collected across different seasons, and DNA metabarcoding was applied to compare the taxonomic composition of dietary items. The results revealed that the wild boar diet included 54 plant genera, 9 animal genera, and 22 macrofungal genera. Among the plants, *Pueraria*, *Quercus*, and *Ipomoea* were the most dominant, with relative abundances of 21.3%, 18.3%, and 16.4%, respectively. The primary animal taxa consisted of *Neomyia* (54.7%) and *Didea* (39.4%), while *Rhizopogon* accounted for 95.3% of the macrofungal sequences detected. Overall, the study demonstrated clear seasonal variation in the diet of wild boars, reflecting their remarkable adaptability to changing environmental conditions.

## Simple Summary

Wild boars are omnivorous animals, and their primary food sources vary based on their habitat. They mainly consume plants, but their diet also includes some animals and macrofungi. This study analyzed the food composition of wild boars during different seasons using metagenomic analysis of fecal samples. The results showed that the diet of wild boars fluctuated by season, with plant sources constituting the majority of their intake, which included 54 different genera. This was followed by macrofungi, comprising 22 genera, and 9 genera of animals. These findings are significant for the management of wild boars and other species in Bukhansan National Park, Republic of Korea. It is crucial to use metagenomic methods to compare and evaluate dietary differences across various ecological regions, and this approach should be integrated into future studies.

## 1. Introduction

Wild boars (*Sus scrofa*) are highly adaptable omnivorous mammals inhabiting diverse ecological zones and exhibiting remarkable dietary flexibility [1]. Instead of consistently consuming preferred food items, wild boars tend to select readily accessible resources to maximize survival [2,3]. Dietary diversity is influenced by habitat conditions and individual body size, with minimal variation between sexes [2,3]. The composition of a diet is closely connected to gut microbiota, and changes in diet can influence the adaptability of this microbiota [4,5]. An analysis of intestinal microbiota from fresh fecal samples in wild boars revealed significant differences that were closely related to the dietary composition in various ecological environments [6].

Previous studies have shown that roughly 90% of the wild boar diet consists of plant material, whereas the remaining 10% comprises animal matter and fungi [1]. The plant-based components commonly consumed by wild boars in their natural habitat include genera such as *Cissus*, *Dioscorea*, *Quercus*, *Actinidia*, and *Houttuynia*, while in agricultural areas, they particularly prefer maize, and the damage caused by wild boars is increasingly significant [2,7,8]. The primary food sources for wild boars include small vertebrates, invertebrates, and animal carcasses, with the main components being *Elaphodus*, *Amynthas*, *Chonaphe*, *Rattus*, and *Tanytarsus* [1,2,9].

The food choices of wild boars are influenced by several factors, which can be grouped into four main categories: the availability of food, energy requirements, seasonal fluctuations, and geographical variations [1]. Seasonal fluctuations strongly influence diet composition; for instance, plant consumption is generally lower in winter than in other seasons. In addition, wild boars consume various plant parts, including seeds, fruits, roots, leaves, and stems, depending on seasonal availability [10]. During winter, wild boars often depend on roots and acorns when food sources above ground are limited. In spring, they prefer the new shoots of herbs [11,12,13]. In summer and fall, their diet typically consists of fruits and agricultural products [8,11]. Although animal-derived foods constitute a smaller fraction of the diet, the seasonal composition of animal food varies; small mammals dominate in autumn and winter, while earthworms are most plentiful in spring and summer [1,14]. Macrofungi that produce large, visible fruiting bodies, such as mushrooms, play a crucial role in forest ecosystems. They contribute to biodiversity and offer nutritional and medicinal benefits [15,16]. Additionally, these fungi serve as important dietary components for many forest-dwelling animals [17,18]. Interestingly, wild boars that are introduced to non-native habitats tend to consume a larger proportion of macrofungi compared to those in their native environments [1]. Furthermore, macrofungi are often present in the diet of wild boars throughout almost all seasons of the year [1].

Studies on wild boar diets have primarily relied on analyses of stomach contents and fecal samples, employing both traditional and modern methodologies [2,19,20]. However, traditional methods such as direct observation or the examination of stomach and fecal contents often face limitations in accurately identifying food remnants due to digestion and decomposition. On the other hand, recent advances in metagenomics, particularly DNA metabarcoding, have enabled more accurate and comprehensive dietary analyses. For instance, a DNA metabarcoding analysis of stomach contents from wild boars in southern China identified 153 plant genera [2]. In Republic of Korea, the diet composition of wild boars (*Sus scrofa coreanus* Heude) was examined using fecal samples collected from Mt. Jeombongsan. This study compared dietary compositions between males and females and among different body sizes and identified the genera *Sanguisorba* and *Filipendula* as dominant plant food sources, whereas the insect orders *Diptera* and *Coleoptera* were detected as animal food components [3].

Despite increasing interest, research on seasonal variations in wild boar diets, including plant, animal, and macrofungi components, remains limited, particularly in Republic of Korea. To address this gap, the present study employed metagenomic techniques to analyze fecal samples collected from wild boars across different seasons in Bukhansan National Park, Republic of Korea. Using DNA metabarcoding, we aimed to characterize and compare the taxonomic composition of plant, animal, and fungal dietary components, thereby providing a comprehensive view of the seasonal foraging ecology of wild boars.

## 2. Materials and Methods

### 2.1. Study Area and Sample Collection

**Figure 1:**
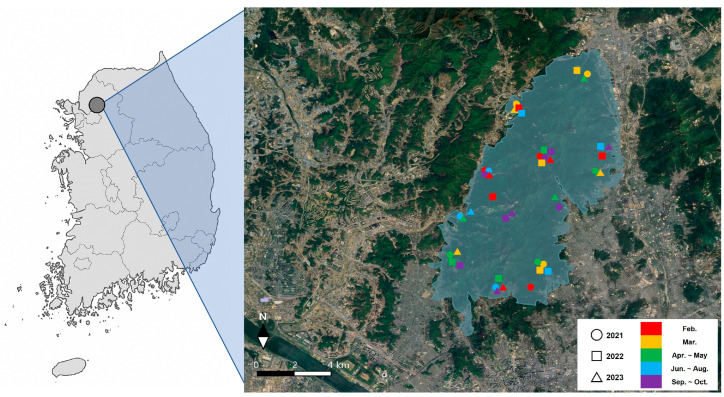
Map of Bukhansan National Park, Republic of Korea, showing the locations of sampling sites where wild boar fecal samples were collected between 2021 and 2023.

All fresh fecal samples were collected at Bukhansan National Park [37.703380, 127.032166], Republic of Korea, during 2021–2023. For each sampling season over three years, three samples were pooled for the analysis of plant-, animal-, and macrofungal-derived dietary components (Figure 1). A total of 45 wild boar fecal samples were analyzed to compare seasonal dietary compositions across five sampling periods: February, March, April–May, June–August, and September–October over the three-year study period. Each fresh fecal sample was carefully scraped from the outer layer, and approximately 20 g was collected and placed in a 50 mL tube. The samples were then transported in a cooler to the laboratory and stored at −20 °C until DNA extraction.

### 2.2. DNA Extraction and PCR Amplification

Fecal samples were thoroughly homogenized before DNA extraction. Genomic DNA was extracted from approximately 250 mg of fecal material using the DNeasy PowerSoil Pro Kit (Qiagen, Hilden, Germany), following the manufacturer’s protocol. A total of 20 μL of purified DNA was eluted from each sample, and DNA from three replicates was pooled before amplification. The quantity and quality control (QC) of the extracted DNAs were assessed using Quant-IT PicoGreen (Invitrogen, Waltham, CA, USA). DNA libraries were constructed using the Illumina metagenomics sequencing library preparation method. To detect plant-derived DNA, the chloroplast *rbcL* gene was amplified using primers rbcLaf (ATGTCACCACAAACAGAGACTAAAGC) and rbcLr509 (AGGGGACGACCATACTTGTTCA) [21]. For animal diet detection, the mitochondrial cytochrome oxidase subunit I (*CO*I) gene was amplified using primers COIF (TYGTYACMGCCCAYGCYTTYGTAATAAT) and COIR (CCTAGAATTGAKGARACACCKGC) [22]. Additionally, the internal transcribed spacer 2 (*ITS*2) region was amplified using primers ITS3 (GCATCGATGAAGAACGCAGC) and ITS4 (TCCGCTTATTGATATGC) to determine macrofungal dietary composition [23]. The initial PCR products were purified using AMPure XP beads (Agencourt Bioscience, Beverly, MA, USA). The purified products were subjected to a secondary PCR to construct sequencing libraries, which were indexed with barcodes using the Nextera XT Index Kit (Illumina, San Diego, CA, USA).

### 2.3. Illumina MiSeq-Sequencing and Bioinformatic Analysis

The length and concentration of amplified products were evaluated using the TapeStation D1000 ScreenTape system (Agilent Technologies, Waldbronn, Germany). The resulting amplicon libraries were sequenced at Macrogen (Seoul, Republic of Korea) using the Illumina MiSeq™ platform (Illumina, San Diego, CA, USA) in accordance with the manufacturer’s protocol. Raw sequences were demultiplexed by index sequences to generate paired-end FASTQ files. Adapter sequences and any used primers were removed with Cutadapt version 3.2 [24]. Additionally, Cutadapt version 3.2 was utilized to trim the forward and reverse reads to specific lengths: 230 bp and 150 bp for plants, 270 bp and 250 bp for animals, and 250 bp for both forward and reverse reads in macrofungi. Error-corrected paired-end sequences were then merged into single contigs, and chimera sequences were eliminated using DADA2 version 1.18.0 to obtain amplicon sequence variants (ASVs) [25]. ASVs shorter than 400 bp (plant), 250 bp (animal) and 140 bp (macrofungi) were filtered out using R version 4.0.3. Comparison analysis of plant, macrofungi, and animal communities was performed using QIIME version 1.9.0 for normalization. Plant taxa were identified using a comparative approach that involved the SINTAX classifier and BLAST version 2.13.0 searches against the NCBI nucleotide non-redundant database, with a query coverage greater than 85% and an identity greater than 85% [26]. Macrofungal communities were classified using the VSEARCH algorithm and the UNITE database, and this process was implemented in ‘vsearch’ version 2.22.1 [27]. Animal taxa were assigned by BLAST searches against the NCBI nucleotide non-redundant database, with a query coverage greater than 85% and an identity greater than 85% [28].

## 3. Results

### 3.1. Plant Diet Composition of Wild Boar in Different Seasons

**Figure 2:**
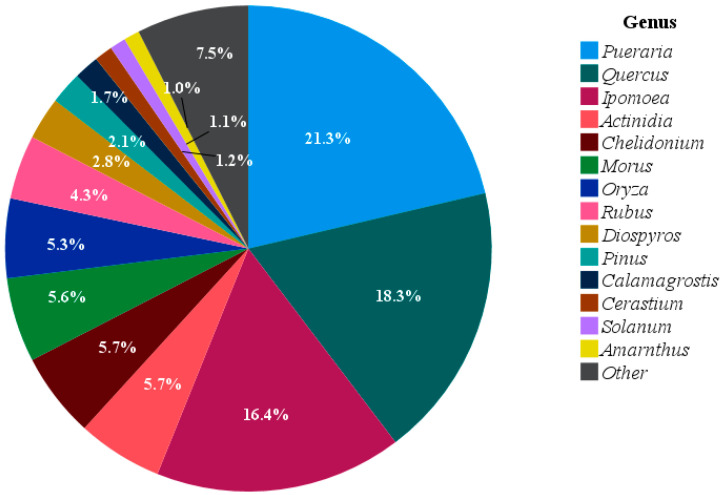
Relative abundance of dominant plant genera detected in all seasonal wild boar fecal samples based on DNA metabarcoding analysis. Plants with percentages below 1% were grouped into the other category.

The wild boar diet was characterized by a wide diversity of plant taxa. A total of 54 plant genera were identified from the dietary analysis. Among them, 14 genera each accounted for more than 1% of the overall plant composition, whereas the remaining 40 genera contributed less than 1% each. The remaining genera were grouped together, collectively representing 7.5% of the total plant composition in the wild boar diet. Among the 14 genera exceeding 1% relative abundance, *Pueraria*, *Quercus*, and *Ipomoea* were the most dominant, contributing 21.3%, 18.3%, and 16.4%, respectively. The remaining genera showed relative abundances ranging from 1.0% to 5.7% (Figure 2).

**Figure 3:**
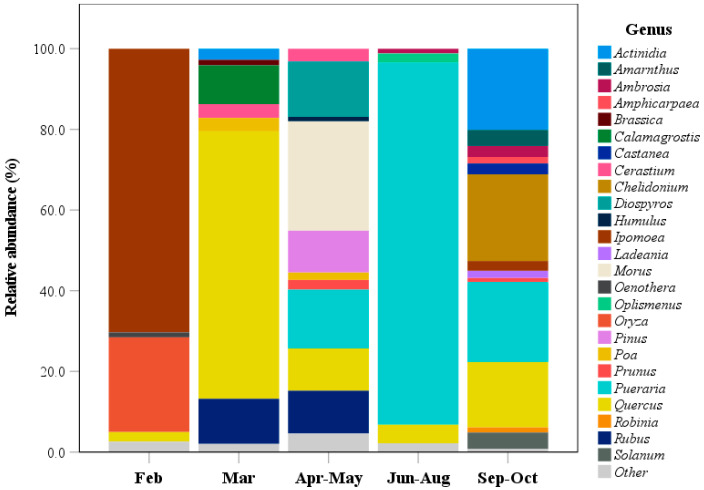
Seasonal variation in the relative abundance of plant genera identified from wild boar fecal samples collected across different seasons using fecal DNA metabarcoding. Plants with relative abundance below 1% were categorized into the other group.

Analysis of relative plant abundances across all surveys indicated that 25 genera exhibited relative abundances greater than 1% (Figure 3). In February samples, only four genera showed a relative abundance greater than 1%. Among these, the genus *Ipomoea* dominated, comprising 70.3% of the plant composition, followed by *Oryza* at 23.5%. *Quercus* and *Oenothera* contributed 2.4% and 1.2%, respectively. In March samples, there were seven genera that exceeded 1% relative abundance. Among these, *Quercus* had the highest relative abundance at 66.3%. *Rubus* and *Calamagrostis* followed, with relative abundances of 11.2% and 9.6%, respectively. The remaining genera, including *Poa*, *Cerastium*, *Brassica*, and *Actinidia*, showed relative abundances between 1.4% and 3.4%. Samples collected during April–May and September–October indicated greater plant dietary diversity during these periods. Ten genera were identified in the April–May samples, whereas 13 genera were identified in the September–October samples. During April–May, *Morus* was the most abundant plant taxon, comprising 27.1% of the total plant composition. During September–October, *Chelidonium* accounted for 21.5% of the plant component of the wild boar diet. Plant composition in samples collected during June–August was primarily dominated by the genus *Pueraria*, which accounted for 89.7% of the total composition. Minor genera such as *Quercus*, *Oplismenus*, and *Ambrosia* accounted for 4.6%, 2.3%, and 1.2%, respectively (Figure 3).

### 3.2. Animal Diet Composition of Wild Boar in Different Seasons

**Figure 4:**
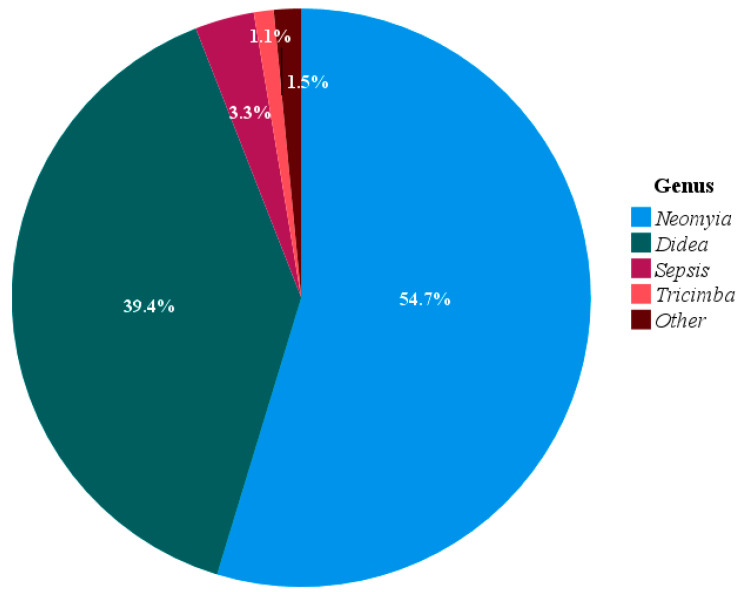
Relative abundance of dominant animal genera detected in all seasonal wild boar fecal samples based on DNA metabarcoding analysis. Animals with percentages below 1% were grouped into the other category.

The animal component of the wild boar diet primarily comprised insects and a few oligochaetes, with 9 genera identified. *Neomyia* accounted for 54.7% of the animal sequences, while *Didea* comprised 39.4%. Additionally, the genera *Sepsis* and *Tricimba* represented 3.3% and 1.1%, respectively. The remaining genera each contributed less than 1% (Figure 4).

**Figure 5:**
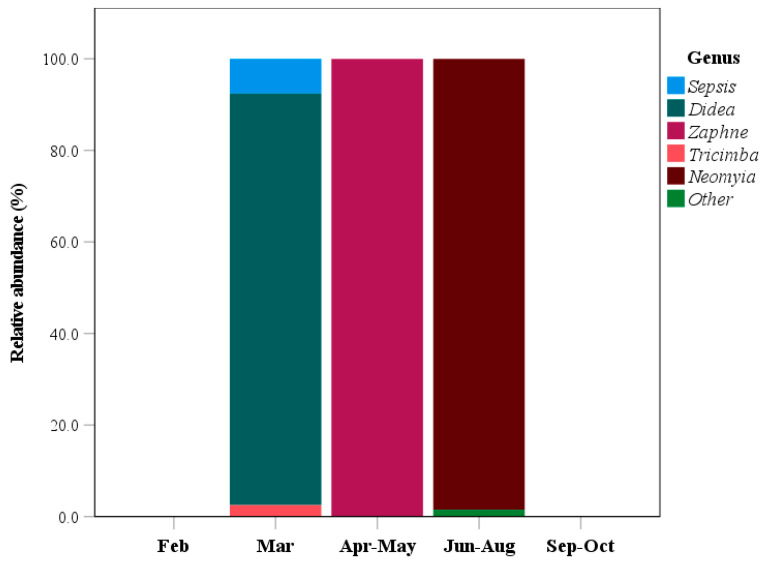
Seasonal variation in the relative abundance of animal genera identified from wild boar fecal samples collected across different seasons using fecal DNA metabarcoding. Animals with relative abundance below 1% were categorized into the other group.

No animal DNA was detected in the samples collected during February and September–October. However, in March samples, three genera, *Didea*, *Sepsis*, and *Tricimba,* were detected, with relative abundances of 89.9%, 7.6%, and 2.5%, respectively. In April–May samples, *Zaphne* accounted for 100% of the animal sequences. During June–August, *Neomyia* dominated, comprising 98.5% of the identified animal sequences. Other genera, including *Stevensonella*, *Eusphalerum*, *Protaetia*, and *Ochthebius*, were detected only in June–August samples, and they had relative abundances of less than 1% (Figure 5).

### 3.3. Macrofungal Diet Composition of Wild Boar in Different Seasons

**Figure 6:**
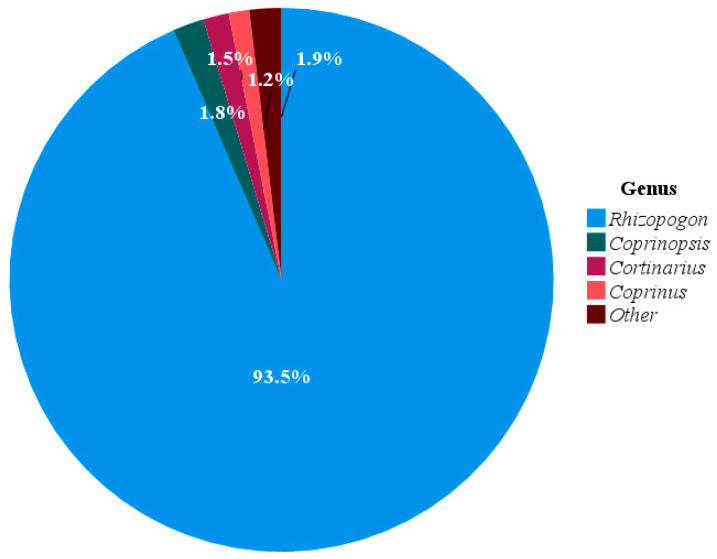
Relative abundance of dominant macrofungal genera detected in all seasonal wild boar fecal samples based on DNA metabarcoding analysis. Macrofungi with percentages below 1% were grouped into the other category.

The composition of macrofungi found in the diet of wild boars was categorized into 22 fungal genera. Among these, *Rhizopogon* was the most prevalent, comprising 93.5% of the total samples investigated. Three other genera with relative abundances greater than 1% were *Coprinopsis* (1.8%), *Cortinarius* (1.5%), and *Coprinus* (1.2%). The remaining genera collectively accounted for 1.9% of the total macrofungal composition (Figure 6).

**Figure 7:**
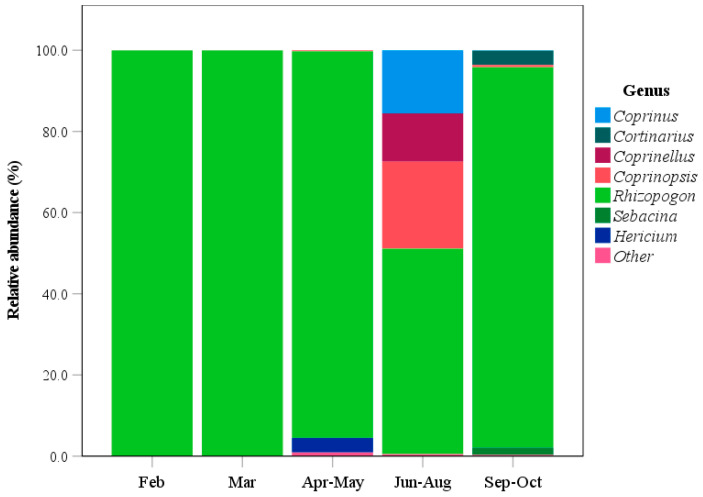
Seasonal variation in the relative abundance of macrofungal genera identified from wild boar fecal samples collected across different seasons using fecal DNA metabarcoding. Macrofungi with relative abundance below 1% were categorized into the other group.

As shown in Figure 7, *Rhizopogon* was the dominant fungal taxon throughout the study period from February to October. In February–March samples, the relative abundance of the genus *Rhizopogon* was approximately 100%. During April–May, *Rhizopogon* comprised 95.3% of the macrofungal sequences, while the genus *Hericium* was also present at a relative abundance of 3.6%. In June–August, the relative abundance of *Rhizopogon* decreased to 50.6%, while several other fungal genera increased in relative abundance. These included *Coprinopsis* at 21.4%, *Coprinus* at 15.5%, and *Coprinellus* at 11.9%. In September–October, the proportion of *Rhizopogon* increased again to 93.7%. Additionally, two other genera with relative abundances greater than 1%, *Cortinarius* at 3.5% and *Sebacina* at 1.8%, were also identified.

## 4. Discussion

Wild boars exhibited a diverse diet composed of 54 plant genera, 9 animal genera, and 22 macrofungal genera (Supplementary Materials). The availability of these dietary resources varied seasonally. Among plant taxa, *Quercus* was detected in all sampling periods, with relative abundances ranging from 2.4% to 66.3% (Figure 3). The composition of animal taxa varied seasonally, with insect taxa showing no overlap among different sampling periods (Figure 5). In terms of macrofungi, *Rhizopogon* was the dominant genus throughout the study period. Seasonal variations in wild boar diet composition have also been documented in previous studies [10,29,30]. For example, an analysis of stomach contents from wild boars in central Punjab, Pakistan, revealed 18 food items in autumn, 23 in winter, 26 in spring, and 15 in summer [10]. Moreover, the proportions of plant and animal materials varied among seasons [10]. In winter, wild boars primarily consumed stems, leaves, and roots, whereas in summer, they fed mainly on seeds, fruits, and tubers [10]. Similarly, in the Czech Republic, the main dietary components of wild boars also exhibited seasonal differences. Acorns and maize were identified as the most significant food sources [29]. Furthermore, natural food sources varied substantially with location and season [29]. During winter, acorns comprised up to 33% of the wild boar diet. This pattern may be attributed to the dormancy of many plant species during winter, leading to leaf loss and limited growth. Consequently, acorns serve as the main available food source and nutritional component for wild boars during this period [29]. Another study from Seoul, Republic of Korea, found significant seasonal variation in the proportion of acorns in the wild boar diet [30]. In spring, acorns accounted for 51.3% of the diet, but this dropped sharply to 0.4% in summer. During winter, their primary nutritional source shifted to plant roots (62.4%), while acorns accounted for only 7.8% [30]. In this study, food derived from *Quercus* showed the highest proportion in March (spring), reaching 66.3%. This finding suggests that acorns likely represent the primary *Quercus*-derived food source. However, further investigation is required to confirm this hypothesis.

The wild boar diet is influenced not only by seasonal factors but also by ecological conditions and body size; however, it appears to be unaffected by sex [3,28,31,32]. DNA metabarcoding analysis of fecal samples from wild boars in the southeastern United States identified 166 plant genera and 18 vertebrate species. The plant component of the diet varied seasonally but remained consistent between males and females. The same study showed that amphibians were the most common vertebrate component in wild boar diets, although mammals, reptiles, and birds were also identified [32]. In this study, we used the *CO*I gene; hence, invertebrates, particularly insects and oligochaetes, constituted the primary animal component of the wild boar diet. Among these taxa, *Neomyia* and *Didea* were the most dominant. The presence of these insect species may be due to insects laying their eggs on the leaves of host trees. When wild boars consume these plants, the eggs could enter their digestive systems. Alternatively, it is also possible that insects find feces after wild boars excrete them and subsequently lay their eggs on the feces. To gain a better understanding of this phenomenon, more detailed studies will be necessary in the future. A previous study examined the abundance of *Neomyia* species in artificial cow dung in the United Kingdom. The dominant species identified were *Neomyia cornicina* and *N. viridescens*, which together comprised up to 58% of the population. That study also reported that environmental factors such as temperature, humidity, and fecal moisture did not significantly affect *Neomyia* abundance [33]. *Didea* larvae are known to be zoophagous and serve as natural enemies; however, their range is projected to decline by over 98% by 2050 [34]. Research on the abundance of *Neomyia* and *Didea* in animal feces remains limited, likely due to a focus on other taxa in previous studies. Consequently, the choice of primer sets in DNA metabarcoding may influence the detection of animal taxa in wild boar diets.

*Rhizopogon* species are hypogeous ectomycorrhizal fungi inhabiting coniferous forests that enhance root absorption of water and nutrients, promote plant growth, and improve resistance to biotic and abiotic stresses [35]. The sporocarps of *Rhizopogon vinicolor* provide essential nutrients for small mammals such as *Clethrionomys californicus* and *Glaucomys sabrinus*, including protein, non-protein, and cell-wall nitrogen [36]. This fungus is consumed by small mammals, including *Clethrionomys californicus* and *Tamias townsendii*. Mature basidiomes of *R. vinicolor* can survive gut passage in these mammals and be isolated from their fecal samples. Furthermore, spores from ingested basidiomes exhibit greater metabolic activity than those from uneaten ones [37]. Hence, spore dispersal in these fungi can partially depend on small mammals [37,38,39]. To accurately assess the role of wild boars in dispersing fungal spores, further research is necessary to examine the frequency of fungal spores found in both their stomachs and intestines. This will help eliminate the possibility of fungal spore contamination occurring after the feces are expelled into the environment. *Rhizopogon* communities differ across various ecoregions and the host plants with which they form symbiotic relationships. A total of 25 *Rhizopogon* species have been reported from both *Pseudotsuga* and *Pinus* forests in the Madrean Sky Islands Archipelago. However, the richness and diversity of *Rhizopogon* communities were higher in *Pseudotsuga* than in *Pinus* forests [38]. Moreover, *Rhizopogon* abundance in mixed forests exceeded that in both hardwood and softwood forests [38]. Truffles constitute an important component of wild boar diets, although their consumption decreases in areas with deep snow [28]. In our study, *Rhizopogon* species accounted for 93.5% of the fungal components, making them a dominant food source throughout all seasons. The relative abundance of these fungi ranged from 50.6% to nearly 100%. In another study, researchers found that wild boars frequently forage for the fruiting bodies of large mushrooms as a food source [40]. Consequently, the presence of fungal material in wild pig feces may result from their consumption of these mushrooms within the food chain. This finding further suggests that wild boars may facilitate the dispersal of *Rhizopogon* spores. However, further studies are needed to isolate and culture these fungi from wild boar feces. Such work would contribute substantially to the conservation and genetic resource development of these symbiotic fungi.

## 5. Conclusions

The diet of wild boars in Bukhansan National Park, Republic of Korea, was highly diverse, comprising plants, animals, and macrofungi. Specifically, plants accounted for 70.0% of the diet, macrofungi for 29.4%, and animals for 0.6%. The diet composition of wild boars varied across seasons. Regarding plant sources, the dominant genera were *Ipomoea*, *Quercus*, and *Pueraria*. *Ipomoea* was primarily consumed in February, *Quercus* was more prevalent in March, and *Pueraria* became the main plant source from June to August. For the animal component, *Neomyia* and *Didea* were dominant. Of these, *Didea* was particularly prevalent in March, whereas *Neomyia* was more abundant during April–May. Although animal-derived foods constituted a minor portion of the diet during June–August, all detected animal sequences belonged to the genus *Zaphne*. *Rhizopogon* was the principal macrofungal taxon in the wild boar diet and was present across all seasons. Overall, wild boars primarily forage for plants and fungi. This behavior highlights the potential for developing effective management strategies that promote the conservation and sustainable use of biological resources in areas surrounding their habitats.

## Data Availability

The data presented in this study are available on request from the corresponding authors. The data are not publicly available due to institutional policy.

## Supplementary Materials

The following supporting information can be downloaded at https://www.mdpi.com/article/10.3390/ani15243598/s1.
